# Factors associated with neurodevelopment in preterm infants with systematic inflammation

**DOI:** 10.1186/s12887-021-02583-6

**Published:** 2021-03-08

**Authors:** Eun Sun Lee, Ee-Kyung Kim, Seung han Shin, Young-Hun Choi, Young Hwa Jung, Sae Yun Kim, Ji Won Koh, Eui Kyung Choi, Jung-Eun Cheon, Han-Suk Kim

**Affiliations:** 1 Department of Pediatrics, Seoul National University Children’s Hospital, 101, Daehak-ro, Jongno-gu, Seoul, 03080 South Korea; 2 Department of Radiology, Seoul National University Children’s Hospital, Seoul, South Korea; 3 Department of Pediatrics, Seoul National University Bundang Hospital, Seongnam, South Korea; 4 Department of Pediatrics, College of Medicine, The Catholic University, Seoul, South Korea; 5 Department of Pediatrics, Soonchunhyang University Cheonan Hospital, Soonchunhyang University College of Medicine, Cheonan, South Korea; 6 Department of Pediatrics, Korea University Ansan Hospital, Korea University College of Medicine, Ansan, South Korea

**Keywords:** Premature, White matter injury, Sepsis, Necrotizing enterocolitis, Inflammation, Amplitude integrated encephalography, Cytokine

## Abstract

**Background:**

Several studies have suggested that adverse neurodevelopment could be induced by systemic inflammation in preterm infants. We aimed to investigate whether preterm infants with systemic inflammation would have impaired neurodevelopment and which biomarkers and neurophysiologic studies during inflammation are associated with poor neurodevelopment.

**Methods:**

This prospective cohort study enrolled infants born before 30 weeks of gestation or with birth weight < 1250 g. Infants were grouped according to the presence of systemic inflammation: Control (no inflammation, *n* = 49), I (systemic inflammation, *n* = 45). Blood and cerebrospinal fluid samples for markers of brain injury and inflammation were collected and amplitude-integrated electroencephalography (aEEG) was performed within 4 h of septic workup. We evaluated aEEG at 35 weeks postmenstrual age (PMA), head circumference at 36 weeks PMA, and brain MRI at discharge. The Bayley Scales of Infant and Toddler Development III (Bayley-III) was performed at a corrected age (CA) of 18 months.

**Results:**

The I group had more white matter injuries (2 vs. 26.7%, Control vs. I, respectively) at the time of discharge, lower brain functional maturation (9.5 vs. 8), and smaller head size (z-score − 1.45 vs. -2.12) at near-term age and poorer neurodevelopment at a CA of 18 months than the control (*p* < 0.05). Among the I group, the proportion of immature neutrophils (I/T ratios) and IL-1 beta levels in the CSF were associated with aEEG measures at the day of symptom onset (D0). Seizure spike on aEEG at D0 was significantly correlated with motor and social-emotional domains of Bayley-III (*p* < 0.05). The I/T ratio and CRP and TNF-α levels of blood at D0, white matter injury on MRI at discharge, head circumference and seizure spikes on aEEG at near-term age were associated with Bayley-III scores at a CA of 18 months.

**Conclusions:**

Systemic inflammation induced by clinical infection and NEC are associated with neurodevelopmental impairment in preterm infants. The seizure spike on aEEG, elevated I/T ratio, CRP, and plasma TNF-alpha during inflammatory episodes are associated with poor neurodevelopment.

## Background

As the survival rate of preterm infants has increased, the management of long-term complications, especially neurodevelopmental impairment, becomes important [1]. The most common brain injury in premature infants is periventricular leukomalacia [2], and its pathogenesis involves the effect of hypoxia, ischemia, and inflammation of the progenitor oligodendrocyte cells present during weeks 23–32 of gestation [3].

Preterm infants are at a high risk for infection, and infection-induced inflammation was found to contribute to adverse neurodevelopment in several clinical and animal studies [4–8]. The mechanisms of systemic inflammation-induced neuronal injury have not been fully elucidated, but systemic inflammation is known to disrupt the blood-brain barrier and incite a local inflammatory response in the brain [7, 9]. The magnitude of systemic inflammation is possibly reflected in the level of blood inflammatory markers and these markers could indicate the high-risk preterm infants for brain injury and poor neurodevelopment at a later stage. A valid diagnostic biomarker needs to be designed for evaluation in a clinical setting of systemic inflammatory state [10–12].. Furthermore, current neonatal intensive care increasingly utilizes non-invasive brain monitors, such as amplitude-integrated electroencephalography (aEEG), to detect acute brain injury, and a previous study showed that aEEG was associated with inflammatory markers and neurodevelopment [13]. However, there have been few studies on the association between adverse neurodevelopment and inflammatory markers or aEEG findings during systemic inflammatory episodes that preterm infants experience during neonatal intensive care unit (NICU) admission.

The objective of this study is two-fold: first, to investigate whether preterm infants who experienced systemic inflammation induced by clinical infection and NEC are more susceptible to impaired short-term neurological and CA of 18 months neurodevelopmental outcomes in our study population, and second, to examine the association of laboratory markers including inflammatory cytokines and brain injury markers and aEEG at the time of systemic inflammation in the NICU and neurodevelopmental outcomes. We hypothesized that inflammation increases cytokine levels and causes brain injury, which are reflected as abnormalities in aEEG and adverse neurodevelopment.

## Methods

### Study design

This was a prospective cohort study. We recruited infants born before 30 weeks of gestation, or whose birth weights was less than 1250 g, and were admitted to the NICU of the Seoul National University Children’s Hospital from December 2013 to June 2017. We excluded infants with congenital anomalies and grade IV intraventricular hemorrhage (IVH).

The subjects were grouped according to the presence of systemic inflammation. During the NICU stay, systemic infection was clinically suspected if there were at least two newly developed signs of fever, apnea, difficulty in breathing, tachycardia or bradycardia, hypotension, irritability, lethargy, and feeding intolerance. For such infants, blood tests, including blood culture, complete blood count (CBC), and C-reactive protein (CRP), were performed subsequently for sepsis evaluation. If at least one of the following abnormal laboratory findings was revealed: (1) elevation of CRP > 1.0 mg/dL, (2) abnormal CBC of elevated proportion of immature neutrophils (I/T ratio ≥ 0.2), or platelets ≤100 × 109/L, and parenteral antibiotics were administered for more than 5 days, they were designated to the systemic inflammation group (I). I group would include subjects who revealed positive blood cultures or were diagnosed with stage II or higher necrotizing enterocolitis (NEC) when they fulfilled aforementioned criteria. If infants did not develop any septic episodes during the NICU stay or show negative laboratory results after the sepsis workup, they were designated to the control group.

In the I group, CBC with differential counts and CRP level were evaluated on day zero (the day of symptom onset), two, and six of the inflammatory episodes along with an aEEG. We performed aEEG within 4 h of septic workup. In addition, plasma and cerebrospinal fluid (CSF) samples were obtained for cytokine and brain injury marker analysis. The CSF tapping was performed for patients suspected of having sepsis or central nervous system infection. For the entire cohort, an aEEG was performed at 35 weeks of postmenstrual age (PMA), a brain MRI was performed before discharge from the NICU, and follow-up information was collected at a corrected age (CA) of 18 months.

To determine whether systemic inflammation is associated with abnormal neurologic or neurodevelopmental outcomes, we compared HC, aEEG, brain MRI, and Bayley scores between the I and control groups. In addition, to identify predictors of neurologic and neurodevelopmental impairment among infants who experienced systemic inflammation, we analyzed the correlation between biomarkers and neurodevelopmental outcomes. This study followed the guidelines set by Declaration of Helsinki and received ethical approval for human subject by the Institutional Review Board of Seoul National University Hospital, and written informed consent was obtained from the parents following a detailed description of the purpose of the study (IRB No. 1301–058-458).

### aEEG

For infants in the I group, aEEG recordings were performed on day 0 (the day of onset of symptoms), 2, and 6 of the inflammatory episodes and at 35 weeks PMA. The infants in the control group were only assessed at 35 weeks PMA. A portable electroencephalogram monitor, Olympic Cerebral Function Monitor (Olympic Medical, Seattle, USA), was used. Continuous impedance was measured, and the electrodes were deemed to be well applied when the impedance was less than 10 kΩ. Continuous recording was performed over 3 h [14–16]. The aEEG scoring system was used to evaluate a maturation score. Four components of the aEEG, including continuity, cycling, amplitude of the lower border, and bandwidth span, were evaluated and the summed score ranged from 0 to 13 [14]. In addition, we classified the aEEG background activity as normal amplitude, the upper margin of band of aEEG activity > 10 μV and the lower margin > 5 μV; moderately abnormal amplitude, the upper margin of band of aEEG activity > 10 μV and the lower margin ≤5 μV; and suppressed amplitude, the upper margin of the band of aEEG activity < 10 μV and lower margin < 5 μV [17]. We monitored the seizure spike at a rapid rise in both the lower and upper margins of the trace, which usually exhibits a ‘saw tooth’ pattern. We also evaluated the simultaneous raw EEG data [18].

### Cytokine and brain injury marker analysis (plasma and CSF)

Plasma samples were collected on the day of symptom onset (day 0). CSF samples were obtained when CSF tapping was performed for patients suspected of having sepsis or CNS infection. The analyses of IL-1 beta, IL-6, IL-8, Myelin Basic Protein (MBP), Tumor Necrosis Factor-alpha (TNF-alpha), S100B, Glial fibrillary acidic protein, and Enolase 2 were performed [19]. The enzyme-linked immunosorbent assay kit (Cloud-Clone Corp, Houston, Texas, USA) and Quantikine® (R&D systems, Minneapolis, Minnesota, USA) were used for the quantitative determination of cytokines and brain injury markers.

### Brain MRI

Brain MRI, including T2 and T1-weighted sequences, was performed at discharge. Experienced pediatric radiologists assessed the degree of white matter injury (WMI) by scoring five categories on MRI, including the nature and extent of white matter signal abnormality, periventricular white matter volume loss, cystic abnormalities, ventricular dilatation, and thinning of the corpus callosum [20]. These five scores were summed and WMI on MRI was categorized as none (total score 5–6), mild (total score 7–9), and moderate to severe (total score 10–15). The radiologists were blinded to the clinical status of the patients.

### Follow-up outcomes at a CA of 18 months

Body measurement data, including weight, length, and head circumference, were collected at a CA of 18 months. The diagnosis of cerebral palsy were collected. The diagnosis of cerebral palsy was made at the discretion of rehabilitation specialists [21]. The patients were assessed using the Bayley Scales of Infant and Toddler Development - the 3rd edition (Bayley-III). The Bayley-III generates composite scores for five domains including cognitive, language, motor, social-emotional, and adaptive behavior [22].

### Statistical analysis

Statistical analyses were performed using the SPSS Statistics 20.0 software (SPSS Inc., Chicago, IL, USA). The chi-squared test was used to compare categorical data between the groups, while the Mann-Whitney U tests were used to compare quantitative data between groups. ANCOVA was used to analyze differences between groups in head circumference, aEEG maturation scores, WMI on MRI, Bayley scores, and incidence of cerebral palsy, while controlling for gestational age (GA), 1- and 5-min Apgar scores, respiratory distress syndrome (RDS), bronchopulmonary dysplasia (BPD), patent ductus arteriosus (PDA) operation, retinopathy of prematurity (ROP) (≥stage III), and IVH (≥Grade III). Pearson’s partial correlation analysis (adjusted for PMA of the inflammatory event and GA) was used to evaluate the relationship between the biomarkers and neurological outcomes including aEEG, brain MRI, and HC in the I group. Correlation analysis (adjusted for PMA of the inflammatory event and GA) was performed to assess the relationship between biomarkers and the results of neurological evaluation, including aEEG, brain MRI, and HC and Bayley scores at a CA of 18 months. *P*-values < 0.05, two-tailed, were considered statistically significant**.**

## Results

### Study population and data collection

**Fig. 1:**
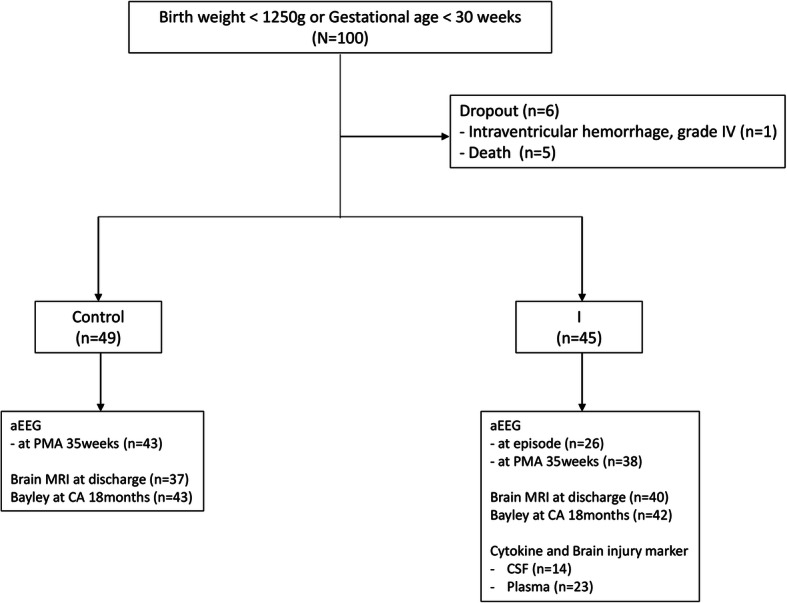
Flow diagram of the cohort study. aEEG, amplitude-integrated electroencephalography; PMA, post menstrual age; CA, corrected age

Of the 100 preterm infants enrolled in the study, five infants who died before 35 weeks of PMA and one infant with IVH grade IV were excluded. There were 49 infants in the control and 45 infants in the I group including 10 infants for culture-proven sepsis, one infant for stage II or higher NEC and one infant for both culture-proven sepsis and stage II or higher NEC. No infants were diagnosed with meningitis. The median PMA at the inflammatory episode were 29^+ 6^ (24^+ 0^–34^+ 6^) weeks, and the median postnatal days of inflammatory episodes with septic work up were 17 (0, 85) days. Nineteen infants had early onset (postnatal days < 7 days) events (Fig. 1).

aEEG at 35 weeks PMA was performed in 43 (88%) infants in the control group and 38 (84%) infants in the I group. aEEG during an inflammatory episode was performed in 26 (58%) infants in the I group. CBC and CRP levels were measured in all infants in the I group. CSF examination was performed in 14 (31%) infants in the I group. Plasma biomarker analyses were performed in 23 (51%) infants on samples collected at symptom onset (day 0), if available. Brain MRI was performed on 37 (75%) and 40 (89%) infants in the control and I groups, respectively. The median age at which brain MRI performed was 37^+ 2^ (33^+ 0^, 62^+ 3^) weeks. In one case, infarction was found on routine brain sonography and MRI was performed at 33 weeks PMA. Bayley test was performed on 43 (88%) infants in the control group and 42 (93%) infants in the I group.

### Perinatal characteristics and neonatal morbidities

The GA (29^+ 2^ vs. 26^+ 6^; Control vs. I respectively if not mentioned otherwise, *p* < 0.001) and birth weight (1090 vs. 770, *p* < 0.001) were lower in the I group than in the control group. In our study, a total of 29 infants (30%) were SGA (14 in the control group and 15 in the I group). There was no significant difference in birth head circumference (z-score) between groups (− 0.49 vs. -0.61, *p* = 0.255). The 1-min and 5-min Apgar scores were significantly lower in the I group and neonatal morbidities including RDS, BPD, PDA operation, and ROP (≥stage III) were significantly more frequent in the I group. (Table 1). Therefore, GA and morbidities were adjusted for the group comparison.

**Table 1:** Perinatal Characteristics, Neonatal Morbidities and Laboratory Result

	Control (***n*** = 49)	I (***n*** = 45)	***P***-value
Gestational age (week)	29^+ 2^ (24^+ 1^, 34^+ 2^)	26^+ 6^ (24^+ 0^, 33^+ 1^)	< 0.001*
Birth weight (g)	1090 (540, 1340)	770 (420, 1400)	< 0.001*
Birth HC (z-score)	−0.49 (− 3.28, 2.55)	− 0.61 (−4.26, 0.98)	*0.255*
Male	20 (40.8)	28 (62.2)	*0.100*
Multiple birth	21 (42.9)	20 (44.4)	*0.877*
Apgar score			
At 1 min	5 (1, 8)	3 (0, 8)	0.028*
At 5 min	7 (1, 9)	7 (0, 9)	0.004*
Cesarean section	33 (67.3)	28 (62.2)	*0.440*
PROM	10 (20.4)	7 (15.6)	*0.541*
HCA	19 (38.8)	21 (46.7)	*0.440*
Preeclampsia	2 (4.1)	3 (6.7)	*0.577*
Antenatal steroid	42 (85.7)	41 (91.1)	*0.294*
SGA	14 (28.6)	15 (33.3)	*0.618*
RDS	26 (53.1)	35 (77.8)	0.012*
Moderate to severe BPD	11 (22.4)	30 (66.7)	< 0.001*
PDA operation	2 (4.1)	14 (31.1)	< 0.001*
ROP ≥ stage III	2 (4.1)	15 (33.3)	< 0.001*
IVH, grade III	0 (0.0)	2 (4.4)	*0.136*
Culture Proven Sepsis	0 (0.0)	11 (24.4)	< 0.001*
NEC ≥ stage II	0 (0.0)	2 (4.44)	< 0.001*
The number of Inflammatory episode		1 (1, 6)	*–*
Postnatal days of Inflammatory episode		17 (0, 85)	*–*
WBC (×10^3^/μl)	–	11.44 (1.00, 39.00)	*–*
I/T ratio	–	0.00 (0.00, 0.89)	*–*
Platelet (×10^3^/μl)	–	157 (30, 811)	*–*
CRP (mg/dL)	–	1.96 (0.01, 18.21)	*–*

Values are presented as median (min, max), or number (%)

*HC* head circumference; *PROM* premature rupture of membranes; *HCA* histologic chorioamnionitis; *SGA* small for gestational age; *RDS* respiratory distress syndrome; *BPD* bronchopulmonary dysplasia; *PDA* patent ductus arteriosus; *ROP* retinopathy of prematurity; *IVH* intraventricular hemorrhage; *I/T ratio* immature to total neutrophil ratio; *CRP* C-reactive protein

* *p* < 0.05

### Short-term neurological and CA of 18 months neurodevelopmental outcome

Head circumference (HC) z-scores at 36 weeks of PMA were significantly smaller in the I group. (− 1.45 vs. -2.12; Control vs. I respectively if not mentioned otherwise, *p* < 0.049). HCs z-scores at a CA of 18 months were also significantly smaller in the I group (0.32 vs. -0.88, *p* < 0.005).

Infants in the I group had significantly more WMI on the MRI at discharge than those in the control group (2 vs. 26.7%, *p* = 0.036). At 35 weeks of PMA, significantly lower maturation scores of aEEG were revealed in the I group (9.5 vs. 8, *p* = 0.017) (Table 2). When comparing Bayley-III scores at a CA of 18 months to those of the control group adjusted for GA, the I group had significantly lower scores in all domains except the social-emotional. However, when further adjusted for neonatal morbidities, only the motor domain showed significant difference (100 vs. 91, *p* = 0.029). There was no significant difference in the incidence of cerebral palsy between the control and the I group (Table 2).

**Table 2:** Short-term Neurological and CA of 18 months Neurodevelopmental Outcome

	Control (***n*** = 49)	I (***n*** = 45)	***P***-value^†^	***P***-value^§^
**HC at PMA 36 weeks (z-score)**	−1.45 (−3.92, 0.51)	−2.12 (−5.28, 0.17)	< 0.001*	0.049*
**aEEG at PMA 35 weeks**				
Maturation score	9.50 (4, 13)	8 (4, 12)	0.002*	0.017*
**WMI on MRI at discharge**			0.008*	0.036*
None	48 (98)	33 (73.3)		
Mild	1 (2)	8 (17.8)		
Moderate to severe	0 (0)	4 (8.9)		
**HC at a CA of 18 months (z-score)**	0.32 (−2.42, 2.36)	− 0.88 (−3.91, 2.13)	< 0.001*	0.005*
**Bayley-III and Cerebral palsy at a CA of 18 months**				
Cognitive	95 (85, 125)	90 (55, 120)	0.006*	*0.076*
Language	97 (74, 121)	89 (50, 121)	0.015*	*0.108*
Motor	100 (82, 118)	91 (46, 110)	0.002*	0.029*
Social Emotional	95 (80, 110)	90 (55, 115)	*0.066*	*0.311*
Adaptive Behaviour	92 (61, 115)	84 (48, 111)	0.005*	*0.055*
Cerebral palsy	2 (4.0)	6 (13.3)	*0.080*	*0.240*

Values are presented as average (min, max), or number (%)

^†^*P*-values are adjusted for gestational age

^§^*P*-values are adjusted for gestational age, Apgar score, RDS, BPD, PDA operation, ROP (≥stage III), and IVH (≥Grade III)

*HC* head circumference; *aEEG* amplitude-integrated EEG; *PMA* Postmenstrual age; *WMI* White matter injury; *MRI* magnetic resonance imaging; *CA* corrected age, *Bayley-III* Bayley Scales of Infant and Toddler Development III

* *p* < 0.05

The patients with culture-proven sepsis or stage II or higher NEC had no significantly different neurologic outcomes when compared with patients with the rest of the I group. In addition, when we compared levels of cytokine, brain injury markers, and CRP and the I/T ratio, there was no significant difference except for higher CRP levels in infants with culture-proven sepsis or stage II or higher NEC.

### Association of aEEG with poor neurodevelopment during systemic inflammation

At the onset of systemic inflammation, there was a significant correlation between I/T ratio and aEEG at the day of symptom onset (D0). When the I/T ratio was high, D0 aEEG showed a lower maturation score (Pearson correlation coefficient with *p*-value, F − 0.496, *p* = 0.026) and more frequent seizure spikes (F − 0.584, *p* = 0.007). CSF IL-1 beta also showed a significant association with the maturation score (F − 0.662, *p* = 0.019), seizure (F − 0.858, *p* = 0.001) and voltage-based classification (F 0.608, *p* = 0.036) in D0 aEEG. Among brain injury markers, MBP showed a significant association with seizure (F − 0.566, *p* = 0.044) (Table 3).

**Table 3:** Correlation between Biomarkers (Day 0) and Neurologic Findings during NICU Admission in the Inflammation Group

	I/T ratio	let	CRP	IL-1 beta		IL-8		TNF alpha		S100B		Enolase2		MBP	
				C	P	C	P	C	P	C	P	C	P	C	P
**aEEG: Day 0**															
Maturation score	−0.496* (0.026)	0.048	0.037	−0.662* (0.019)	0.055	− 0.264	0.063	0.240	−0.169	− 0.130	0.115	− 0.247	0.224	− 0.101	0.018
Seizure^a^	−0.584* (0.007)	−0.086	0.080	−0.858* (0.001)	0.215	0.092	0.015	0.220	−0.405	0.253	0.056	0.208	0.199	0.042	−0.566* (0.044)
Voltage based^b^	0.401 (0.052)	−0.094	−0.092	0.608* (0.036)	0.281	0.138	0.744	−0.173	0.147	−0.019	0.183	0.049	−0.148	0.049	−0.012
**aEEG: PMA 35 weeks**															
Maturation score	−0.621 (0.055)	0.314	−0.208	−0.278	− 0.109	0.046	− 0.280	−0.338	− 0.252	−0.013	0.321	−0.012	0.075	−0.056	− 0.148
Seizure^a^	0.172	−0.376	−0.597	0.312	1.000	0.219	−0.417	0.256	0.179	−0.053	0.179	0.116	−0.030	0.219	0.362
Voltage based^b^	0.085	− 0.295	0.359	0.021	0.030	0.037	−0.253	0.391	0.474	0.200	0.310	0.146	0.249	0.174	0.425
**HC: 36 weeks (cm)**	0.048	0.283	−0.1	0.010	− 0.405	− 0.742* (0.014)	0.408	− 0.771* (0.009)	− 0.493	− 0.792* (0.006)	0.174	− 0.729* (0.017)	− 0.725* (0.018)	− 0.286	− 0.405
**Brain WMI at discharge**^c^	0.483* (0.031)	− 0.027	− 0.098	0.426	−0.185	− 0.104	0.128	− 0.073	−0.195	− 0.278	− 0.374	− 0.303	−0.007	− 0.122	0.077

Day 0: the day of inflammatory symptom onset

The values are Pearson correlation coefficient. If the p-value is less than 0.05, with (p-value). All values are adjusted for gestational age and postmenstrual age of inflammatory event

^a^Seizure (1: yes, 2: no), ^b^Voltage based classification (1: normal, 2: moderate abnormal, 3: severe abnormal), ^c^Brain WMI (1: normal, 2: mild, 3: moderate to severe)

*C* Cerebrospinal fluid; *P* plasma; *MBP* Myelin Basic Protein; *TNF alpha* Tumor Necrosis Factor-alpha; *aEEG* Amplitude-integrated electroencephalography; *PMA* postmenstrual age; *HC* head circumference; *WMI* white matter injury

* *p* < 0.05

Seizure spike on D0 aEEG had a significant correlation with motor (F 0.581, *p* = 0.007) and social-emotional (F 0.544, *p* = 0.013) domains of Bayley-III at a CA of 18 months (Table 4).

**Table 4:** Correlation between Neurologic Markers during Admission and Neurodevelopment (CA of 18 months) in the Inflammation Group

	aEEG at Day 0			aEEG at PMA 35 weeks			Brain WMI^¶^ at discharge	Head circumference at 36 weeks
	Maturation score	Seizure spike^a^	Voltage base^b^	Maturation score	Seizure spike^a^	Voltage base^b^		
**Bayley score**								
Cognitive	0.124	0.040 (0.052)	− 0.269	0.283	0.376* (0.034)	−0.133	− 0.371* (0.020)	0.193
Language	−0.127	0.171	−0.102	0.246	0.408* (0.021)	−0.293	− 0.271	0.210
Motor	0.141	0.581* (0.007)	−0.269	0.161	0.227	0.030	−0.431* (0.006)	0.345* (0.032)
Social emotional	0.024	0.544* (0.013)	−0.175	0.206	0.356* (0.045)	−0.098	− 0.350* (0.029)	0.177
Adaptive behaviour	0.152	0.371	−0.288	0.454* (0.009)	0.229	**−0.349 (0.050)**	− 0.334* (0.038)	0.368* (0.021)
HC at a CA of 18 months	− 0.092	0.031	− 0.002	0.177	–	− 0.174	− 0.167	0.740* (< 0.001)

Day 0: the day of inflammatory symptom onset

The values are Pearson correlation coefficient. If the *p*-value is less than 0.05, with (*p*-value)

All values are adjusted for gestational age and postmenstrual age of inflammatory event

^a^Seizure (1: yes, 2: no), ^b^Voltage based classification (1: normal, 2: moderate abnormal, 3: severe abnormal), ^¶^Brain WMI (1: normal, 2: mild, 3: moderate to severe)

*aEEG* amplitude-integrated EEG; *PMA* Postmenstrual age; *WMI* White matter injury; *HC* Head circumference

* *p* < 0.05

### Biomarkers during systemic inflammation associated with poor neurodevelopment

In the correlation analysis, the higher the D0 I/T ratio, the more WMI in brain MRI at discharge (F 0.483, *p* = 0.031). Several CSF biomarkers ​​showed a significant negative correlation with HC at 36 weeks of PMA: IL-8 (F − 0.742, *p* = 0.014), TNF-alpha (F − 0.771, *p* = 0.009), S100B (F − 0.792, *p* = 0.006), and Enolase 2 (F − 0.729, *p* = 0.017). Among plasma samples, only Enolase-2 presented a negative correlation with HC at 36 weeks. There was no significant correlation between biomarkers and aEEG parameters at 35 weeks PMA (Table 3).

I/T ratio was negatively correlated with the motor (F − 0.530, *p* = 0.016) and social-emotional (F − 0.467, *p* = 0.038) domains in Bayley-III at a CA of 18 months. D0 CRP was negatively correlated with the language (F − 0.330, *p* = 0.033) and motor (F − 0.330, *p* = 0.033) domains. Among the biomarkers, plasma TNF-alpha particularly showed a significant correlation with most domains of Bayley-III: the cognitive (F − 0.662, *p* = 0.037), the motor (F − 0.749, *p* = 0.013), and the adaptive behavior (F − 0.783, *p* = 0.007). In addition, CSF IL-1 beta showed a significant negative correlation with the social-emotional domain (F − 0.764, *p* = 0.016) (Table 5). In correlation analysis, IL-6 was not significantly associated with neurological outcomes.

**Table 5:** Correlation between Biomarkers (Day 0) and Neurodevelopment (CA of 18 months) in the Inflammation Group

	I/T ratio	let	CRP	IL-1 beta		IL-8		TNF alpha		S100B		Enolase2		MBP	
				C	P	C	P	C	P	C	P	C	P	C	P
**HC: CA of 18 months (z-score)**	−0.018	0.200	− 0.032	− 0.110	− 0.388	−0.343	0.403	−0.562	− 0.792* (0.006)	−0.429	− 0.216	−0.326	− 0.763* (0.010)	−0.172	− 0.309
**Bayley score: 18 months of CA**															
Cognitive	−0.307	0.062	−0.294	−0.604	− 0.086	0.246	− 0.255	0.141	− 0.662* (0.037)	0.127	0.134	0.209	−0.090	−0.287	− 0.352
Language	−0.032	0.102	−0.204 (0.050)	− 0.270	−0.133	0.208	−0.270	− 0.117	−0.617* (0.058)	0.049	−0.151	0.177	−0.261	− 0.319	−0.328
Motor	−0.530* (0.016)	0.101	−0.330* (0.033)	−0.649 (0.058)	− 0.005	0.067	− 0.221	−0.052	− 0.749* (0.013)	0.008	0.267	0.100	−0.144	−0.120	− 0.445
Social emotional	−0.467* (0.038)	0.019	−0.222	− 0.764* (0.016)	0.383	0.420	−0.098	0.395	−0.452	0.554	−0.191	0.533	−0.252	− 0.152	−0.632 (0.050)
Adaptive	−0.325	0.119	−0.300	−0.405	− 0.186	0.202	− 0.083	−0.161	− 0.783* (0.007)	0.140	− 0.043	0.255	− 0.295	−0.074	− 0.504

Day 0: the day of inflammatory symptom onset

The values are Pearson correlation coefficient. If the p-value is less than 0.05, with (*p*-value). All values are adjusted for gestational age and postmenstrual age of inflammatory event

*C* Cerebrospinal fluid; *P* plasma; *MBP* Myelin Basic Protein; *TNF alpha* Tumor Necrosis Factor-alpha; *HC* head circumference; *CA* corrected age

* *p* < 0.05

### Head circumference and neurodevelopment at a CA of 18 months

After adjusting for the GA and PMA of the inflammation episode, the HC at 36 weeks of PMA and at a CA of 18 months were correlated (F 0.740, *p* < 0.001). In addition, there was a significant positive correlation between the HC at 36 weeks of PMA and the Bayley scores (motor, F 0.345, *p* = 0.032; adaptive behavior, F 0.368, *p* = 0.012) (Table 4).

## Discussion

Preterm infants who experienced systemic inflammation in the NICU had smaller head sizes and abnormal functional brain maturation at near-term age, more WMI at discharge, and had poor neurodevelopment at a CA of 18 months. In addition, the analyses to find associated factors for poor neurodevelopment revealed that elevated levels of I/T ratio, CRP, and plasma TNF-alpha and the presence of seizure spikes on aEEG at the initial phase of inflammatory episodes were correlated with neurodevelopmental outcomes. Our study is the first to comprehensively evaluate head growth, blood markers, aEEG, brain MRI, and neurodevelopment at a later stage in premature infants who experienced systemic inflammation.

The HC at 36 weeks of PMA was smaller in the I group compared to that in the control group. The group differences in HC were maintained at a CA 18 months after adjusting for GA. The HC at 36 weeks of PMA also correlated with Bayley scores at a CA of 18 months. Therefore, smaller head size at 36 weeks of PMA shown in the I group could be an indicator of poor neurodevelopment at a later stage.

Infants in the I group had significantly more WMI on the conventional MRI at discharge than those in the control group. A recent study using diffusion MRI reported differences in the white matter microstructure of the corpus callosum of 6-year-old preterm children in relation to the presence or absence of severe inflammatory conditions in the neonatal period [23]. The authors concluded neonatal inflammation is one medical factor that may contribute to variation in long-term neurobiological and neuropsychological outcomes in preterm infants.

Compared to the control group after adjusting for GA, the I group had significantly lower cognitive, language, motor, and adaptive behavior scores. However, when the morbidities were adjusted as well, only the motor domain remained significant. Previous studies suggested that preterm infants with postnatal sepsis or NEC are more likely to have adverse neurodevelopment [4, 5, 7, 24, 25]. In our study, infants with systemic inflammation without proven sepsis or NEC also had delayed maturation on aEEG, more WMI, and adverse CA of 18 months neurodevelopmental outcomes indistinguishable from infants with proven sepsis or NEC; this has been inconsistently shown in previous studies. Some studies reported that clinical infection without proven sepsis was also associated with poor neurodevelopment or brain white matter abnormalities [4, 26], but in another study, the major impact on neurodevelopmental outcomes was confined to preterm infants with proven sepsis [7].

We attempted to identify biomarkers associated with a high risk for neurodevelopmental impairment among infants with systemic inflammation. First, we analyzed whether the aEEG was associated with poor neurodevelopmental outcomes. The seizure spike on D0 aEEG had significant relationship with the motor and social-emotional domains. The increase in I/T ratio and CSF IL-1 beta, which are the indices of systemic and central nervous system inflammation, respectively, was significantly related to an increased seizure spike and low maturation of D0 aEEG. This finding implies that abnormal aEEG findings at symptom onset might be mechanistically linked to the inflammatory status in infants. Septic adult patients without central nervous system infection develop diffuse cerebral dysfunction and show characteristic EEG findings, known as sepsis-associated encephalopathy [27]. Preterm infants also show acute electroencephalographic changes such as burst suppression during sepsis [15].

Second, blood markers during inflammatory episodes were analyzed. It is well known that an increased I/T ratio and CRP and thrombocytopenia suggest infection [28, 29]. D0 CRP was negatively correlated with the language and motor domains, and the D0 I/T ratio was negatively correlated with the motor and social-emotional domains. Several studies have shown that brain injury is associated with high inflammatory cytokine levels [30–32]. We analyzed inflammatory cytokines and brain injury markers from D0 plasma and CSF. CSF IL-8, TNF-alpha, S100B, and Enolase 2 showed negative correlations with HC at 36 weeks of PMA; however, they were not correlated with HC at a CA of 18 months or Bayley scales. In a previous study, concentrations of inflammation-related proteins including IL-6, TNF-R2, IL-8, MCP-1, and ICAM-1 in plasma were associated with the risk of microcephaly at a CA of 2 years [33].

Unlike other plasma markers, plasma TNF-alpha was correlated with scores of many domains in Bayley scales and HC at a CA of 18 months. On the other hand, CSF TNF-alpha was not associated with neurodevelopment and only correlated with HC at 36 weeks of PMA as with other CSF cytokines and brain injury markers. This finding might imply the critical role of plasma TNF-alpha in the mechanism of inflammation-associated brain injury in preterm infants. In the Extremely Low Gestational Age Newborn (ELGAN) study, elevated levels of TNF-alpha in blood collected on postnatal day 14 are associated with impaired mental and motor development at 2 years of age [34]. From the same cohort, preterm infants who had sustained elevations of inflammation-related proteins including CRP, TNF-alpha, and IL-8 in the first postnatal month are reported to be more likely to have cognitive impairment at 10 years of age [35]. While the ELGAN study collected cytokine samples independent of inflammatory events, our study analyzed the cytokines from samples obtained at the time of systemic inflammation. We think our approach has the potential to set an appropriate timing for collecting samples when we investigate the effect of systemic inflammation on brain injury and subsequent neurodevelopment because blood-brain barrier disruption is known to be a late or terminal event seen in large-scale activations of the immune system, such as sepsis [9].

From the above results, aEEG findings and blood biomarkers during inflammatory episodes were associated with poor neurodevelopment. Interestingly, the neurodevelopmental outcomes were more closely associated with usual laboratory markers of I/T ratio or CRP rather than most of the inflammatory cytokines or brain injury markers investigated in this study. Notably, this is an observational cohort study; therefore, causation of study findings cannot be concluded, although there were some significant correlations between aEEG, CRP, and plasma TNF-alpha during inflammation and late-stage Bayley scores.

Our study has several strengths. We attempted to capture the point of significant systemic inflammation and conducted a comprehensive evaluation encompassing growth, body fluid biomarkers, neurophysiologic study, brain imaging, and CA of 18 months neurodevelopmental outcomes. We performed Bayley-III, which provides not only granular evaluation of cognitive, language, and motor development, but also social-emotional and adaptive behavior. This has enabled us to examine more diverse aspects of development in study infants. Limitations of this study are the sample size is modest from a single center, and aEEG and cytokine analysis during inflammation were partly performed in the I group. The D0 may not be the first day of an inflammatory episode for infants exposed to chorioamnionitis because sepsis workup was performed only when symptoms of systemic infection were present.

## Conclusion

Our prospective cohort study showed that systemic inflammation induced by clinical infection and NEC is associated with poor neurodevelopment in preterm infants. The seizure spike on aEEG and elevated I/T ratio, CRP, and plasma TNF-alpha during inflammatory episodes are associated with poor neurodevelopmental outcomes among preterm infants who were exposed to systemic inflammation. These results may help estimate the neurodevelopmental risk of individual patients who have inflammatory illness and narrow down the target population of neuroprotection in the future.

## Data Availability

All data generated or analysed during this study are included in this published article.

## Abbreviations

aEEG: Amplitude-integrated electroencephalography
NICU: Neonatal intensive care unit
IVH: Intraventricular hemorrhage
CBC: Complete blood count
CRP: C-reactive protein
CSF: Cerebrospinal fluid
PMA: Postmenstrual age
CA: Corrected age
MBP: Myelin Basic Protein
TNF-alpha: Tumor Necrosis Factor-alpha
WMI: White matter injury
Bayley-III: Bayley Scales of Infant and Toddler Development - the 3rd edition
GA: Gestational age
RDS: Respiratory distress syndrome
BPD: Bronchopulmonary dysplasia
PDA: Patent ductus arteriosus
ROP: Retinopathy of prematurity
NEC: Necrotizing enterocolitis
HC: Head circumference
